# The risk of depressive and anxiety symptoms in women with premature ovarian insufficiency: a systematic review and meta-analysis

**DOI:** 10.1007/s00737-022-01289-7

**Published:** 2023-01-27

**Authors:** Dan Xi, Biyin Chen, Hui Tao, Yunxiang Xu, Guizhen Chen

**Affiliations:** 1 Shenzhen Bao’an Traditional Chinese Medicine Hospital, Guangzhou University of Chinese Medicine, Shenzhen, 518101 Guangdong China; 2 Clinical Medical College of Acupuncture, Moxibustion and Rehabilitation, Guangzhou University of Chinese Medicine, Guangzhou, 510405 Guangdong China

**Keywords:** Premature ovarian insufficiency, Anxiety, Depression, Meta-analysis

## Abstract

Premature ovarian insufficiency (POI) appears to be associated with depressive and anxiety symptoms. However, there is a lack of high-quality evidence relating to the risk of patients with POI developing depression or anxiety. Therefore, we conducted a systematic review and meta-analysis to quantify the risk of depressive and anxiety symptoms in women with POI. We searched English and Chinese databases to evaluate the risk of depression and anxiety disorders in patients with POI. The final search date was November 2021. The risk was quantified using meta-analysis, with an estimation of pooled odds ratio (OR) and 95% confidence interval (CI). Sources of heterogeneity were explored by subgroup analysis. A total of seven primary studies with 1316 individuals were included, five of which were related to depression and six to anxiety disorders. All included articles were case–control studies of high quality. Patients with POI were associated with a higher odds of depression and anxiety (depression: OR = 3.33, 95% CI = 2.31–4.81, *P* < 0.001; anxiety: OR = 4.89, 95% CI = 3.28–7.30, *P* < 0.001). Subgroup analysis also indicated that patients with POI are at a higher risk of anxiety and depression. POI appears to be associated with a high risk of depression and anxiety. Early psychosocial assessment and regular screening of patients with POI are also necessary. In addition, it is important to consider the mental health of patients with POI.

**Supplementary Information:**

The online version contains supplementary material available at 10.1007/s00737-022-01289-7.

## Introduction

Premature ovarian insufficiency (POI) is characterized by menstrual disturbances along with high gonadotropin and low estrogen levels. The prevalence of POI is approximately 1–3% in the general female population (Webber et al. 2016). However, the etiology of POI is still unclear (Ishizuka 2021). Ovarian failure is often irreversible in patients diagnosed with POI. Moreover, the decline of ovarian function in these patients is accompanied by physical, psychological, and psychiatric symptoms (Podfigurna-Stopa et al. 2016). It has been shown that patients with premature ovarian failure (POF) have a significantly higher risk of developing anxiety, depression, sensitivity, hostility, and psychological problems than healthy controls (Van der Stege et al. 2008). Anxiety and depressive symptoms are the most commonly seen mood disorders. These physical symptoms and psychiatric disorders seriously affect the well-being of patients.

Mental disorders in women with POI are often overlooked in a clinical setting. Patients usually emphasize infertility and menstrual problems while neglecting psychological symptoms. In addition, physicians also tend to ignore the psychological problems of these patients. One-third of people with POI who suffer moderate-severe anxiety or depression are underdiagnosed, according to a cross-sectional study (Menezes et al. 2020). The low treatment rates and poor clinical outcomes are primarily due to the poorer awareness of these patients with regard to psychiatric disorders. Therefore, it becomes more challenging to identify a patient’s psychological symptoms in clinical practice (Cuijpers et al. 2011).

Mental illness may also affect fertility and treatment outcomes. In a previous study, Ventura et al. found that there was a positive correlation between mental status and ovarian function status in patients with POI (Ventura et al. 2007). Anxiety and depressive disorders are common psychological disorders, which usually occur together. A previous survey showed that 84% of older adults with depression (including subthreshold depression) also suffered from anxiety or subthreshold anxiety; furthermore, 54% of those with anxiety also had a depressive disorder (Prina et al. 2011). Consequently, it is important to attract more attention to anxiety or depressive symptoms in patients with POI in clinical practice to prevent and treat psychological disorders and improve the patient’s quality of life.

Little is known about the anxiety or depressive symptoms associated with POI. Most studies on anxiety or depressive symptoms in patients with POI are observational. There are no quantitative or qualitative studies on this topic. The meta-analysis approach improves statistical accuracy by integrating multiple studies in a quantitative manner, thus improving objectiveness and comprehensiveness. Therefore, we performed a systematic review and meta-analysis on the risk of depression and anxiety in females with POI. We hope that our findings will raise awareness among physicians with regard to the significance of psychiatric screening and treatment for women with POI, as well as providing evidence-based medical data to assist with clinical decision-making.

## Methods

This report is written in accordance with the MOOSE statement (Stroup et al. 2000) and PRISMA guidelines (Moher et al. 2009). This systematic review and meta-analysis had been pre-registered with PROSPERO (CRD 42,021,285,245).

### Retrieval strategy

Two researchers independently searched electronic databases and ultimately cross-checked them. We searched four English medical databases and four Chinese databases (Cochrane Library, EMBASE, PubMed, Web of Science, Wan Fang Data, China National Knowledge Infrastructure (CNKI), Chongqing VIP Information (CQVIP)). We searched the grey literature including the Chinese Clinical Trial Registry (https://www.chictr.org.cn/historyversionpuben.aspx) and ClinicalTrials.gov (https://www.clinicaltrials.gov/). Only papers published in English and Chinese were searched for from the inception of the databases to October 2021. Medical subject headings and free word combinations search (“Primary Ovarian Insufficiency”; “Premature Ovarian Failure”; “Ovarian Failure, Premature”; “Premature Menopause”). The researcher systematically learned the methods for searching the literature and evaluating the quality of studies.

The specific literature search strategy is as follows: Firstly, subject terms and free terms were identified; secondly, all search results were aggregated after performing a comprehensive search; thirdly, ineligible literature would be eliminated after reading the titles and abstracts of the literature. For the controversial parts, a third reviewer was involved and made the decision. The complete strategies and evidence of retrieval are in Appendix 1.

### Inclusion and exclusion criteria

The inclusion criteria for the article were as follows: (1) type of article—observational studies regarding POI and the risk of prevalence of anxiety or depression; (2) participants were women under 40 years old with POI and symptoms of anxiety or depression; (3) comparisons—the patients with POI were compared with a control group population without POI; and (4) outcomes—prevalence or odds ratio (OR) of depression or anxiety in POI with 95% confidence interval (CI) or calculated ORs with 95% CIs. Anxiety and depression were measured by self-report or physician diagnosis.

The exclusion criteria for the article were as follows: (1) a sample size of fewer than 30 cases; (2) lack of a control group, inappropriate statistical methods, or failure to obtain ORs and 95% CIs; (3) the original literature did not report clear methods of study or the quality of the literature was low, and (4) the literature is duplicated or for which no original text or data is available (Table 1).

**Table 1:** Characteristics of included studies in the meta-analysis

References	Studies design	Sample size(*n*)	Diagnosis method	OR and CI of anxiety	OR and CI of depression	Region	Adjust of confounders	NOS scores
Xilu and Jing 2015	Case-control study	160	STAI	6.178(1.024−41.683)	NA	China	NA	7
Yan and Qin 2015	Case-control study	300	Self-reported	3.875(1.402−5.899)	3.875(1.402−5.899)	China	Age, residence, economic income	7
Wei 2015	Case-control study	98	Self-reported	2.571(0.878−7.534)	1.887(0.571−6.241)	China	NA	7
Qun 2016	Case-control study	140	Self-reported	NA	2.4(1.215−4.740)	China	Marital and childbearing history, past medical history, surgical history, life habits, behavior	8
Lihong 2016	Case-control study	336	STAI	4.5(1.930−10.493)	NA	China	Age, residence	9
Yixuan et al. 2019	Case-control study	123	SAS/SDS	12.73(4.12−39.37)	9.38(3.31−26.57)	China	NA	8
Davis 2010	Case-control study	159	CES-D/STAI	6.672(2.448~18.181)	3.072(1.425−6.622)	USA	Illness uncertainty， purpose in life，stigma，goal re-engagement	7

*CES-D* Center of Epidemiologic Studies Depression Scale; *STAI* the state anxiety subscale of the State-Trait Anxiety Inventory; *SAS* Self-rating Anxiety Scale; *SDS* Self-rating depression scale; *OR* odds ratio; *CI* confidence interval; *NA* not acquire

### Data extraction

Literature screening and data extraction were performed independently by two researchers. First, Endnote (version X9.0) is used to remove duplicate publications from each database. Irrelevant literature was removed by reading the paper titles and abstracts. Second, the literature was re-screened by reading the full text. From each included study, we extracted data containing authors, year of publication, region, number of samples, type of study, features of confounding factors, and diagnostic criteria of study subjects (Table 2). In case of disagreement, the decision was made by a third investigator. If additional data are needed, one may contact authors by email and phone. Two articles did not provide the corresponding data. We contacted the authors of the articles and have not yet received a response.

**Table 2:** Results of subgroups analyses of depression-related ORs and 95% CIs

Total and subgroups	Studies	Heterogeneity (*I*^*2*^*, P*)	Model	OR (95% CI)	*P*
Total	5	31, 0.22	Fixed	3.33(2.31−4.81)	< 0.001
Assessment of depression					
CES-D	1	/	Fixed	3.07(1.42−6.62)	0.004
SDS	1	/	Fixed	9.38(3.31−26.57)	< 0.001
Self-report	3	0, 0.5	Fixed	2.81(1.78−4.44)	< 0.001
Ethnicity					
America	1	/	Fixed	3.07(1.42−6.62)	0.004
China	4	48, 0.13	Fixed	3.41(2.25−5.19)	< 0.001
Adjustment of confounding factors					
Yes	3	0, 0.64	Fixed	3.03(2.00−4.59)	< 0.001
No	2	75, 0.05	Random	4.33(0.90−20.82)	0.07

*CES-D* Center of Epidemiologic Studies Depression Scale; *SDS* Self-rating depression scale; *OR* odds ratio; *CI* confidence interval

### Quality assessment

Two investigators independently evaluated the quality of the literature and cross-checked the results. When there was a dispute, the third author was involved in the appraisal process. Case–control studies were evaluated by using the Newcastle Ottawa Scale (NOS scale) (Wells 2014), low-quality studies (0–3 points), moderate studies (4–6 points), and high-quality studies (7–9 points). Cross-sectional studies were evaluated by using the Agency for Healthcare Research and Quality (AHRQ).

### Data pooling and analysis

The odds ratios (ORs) were used as the effect indicator and 95% CIs, and log (OR) values were calculated. Data integration was performed by using RevMan (version 5.4). Heterogeneity was tested by Cochrane’s *Q* test and *I*^2^ statistic. Heterogeneity (*I*^2^) was considered low at 25%, moderate at 50%, and high at 75%. The fixed effects model is selected if* P* > 0.10 and *I*^2^ > 50%. The random-effects model is applied if *P* ≤ 0.10 and *I*^2^ > 50%, and heterogeneity among included studies was considered.

### Publication bias and sensitivity analysis

A funnel plot was used to assess the publication bias qualitatively. Sensitivity analysis was conducted by removing individual studies one by one, to determine the extent to which each study affected the combined effect. Furthermore, subgroup analyses were also conducted to analyze the possible sources of heterogeneity and assess the association between POI and depression or anxiety.

## Results

### Basic characteristics and the quality of the studies

**Fig. 1:**
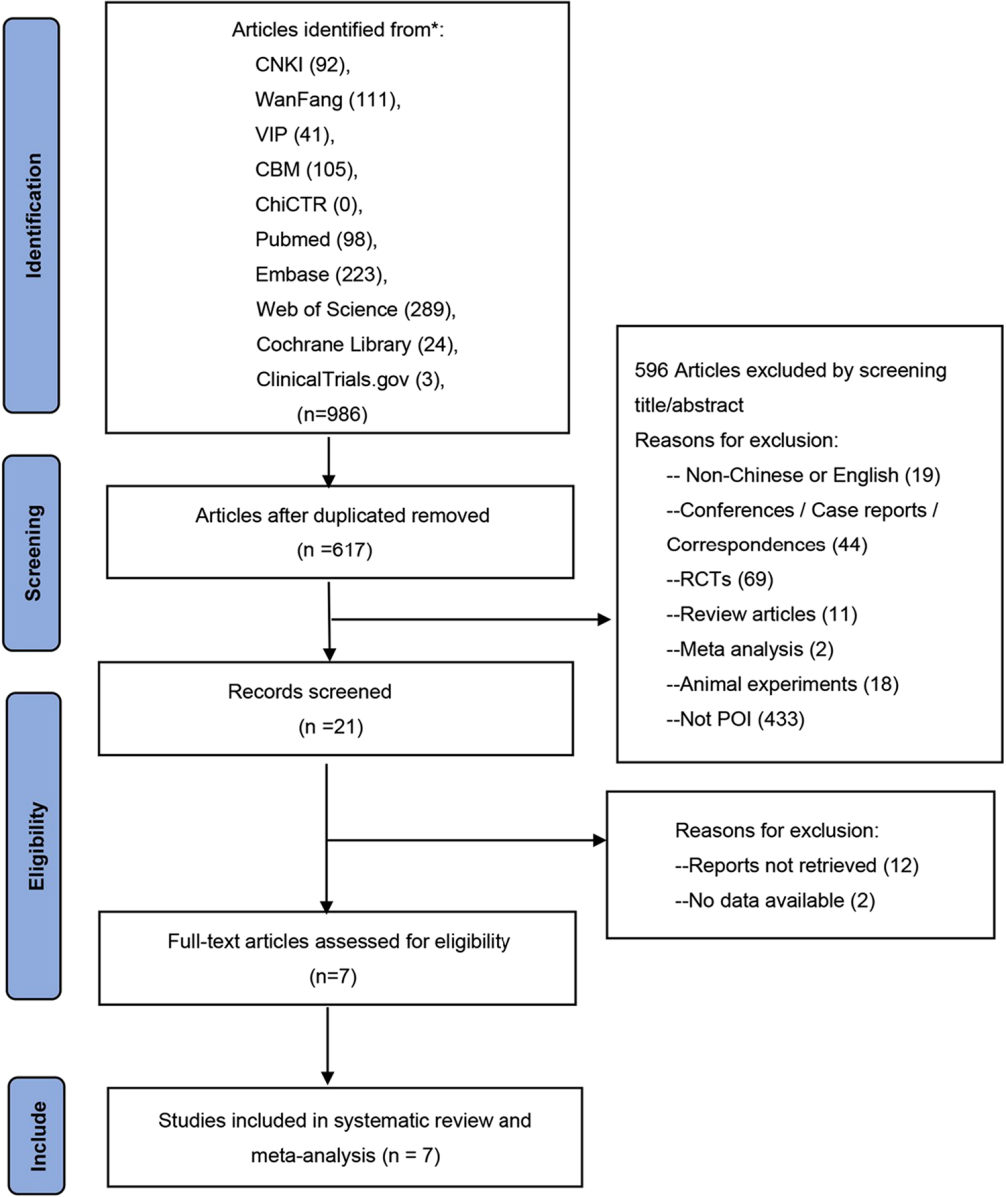
Study selection flow diagram

Figure 1 shows the procedure used to screen the literature. A total of 986 publications were retrieved, and seven studies (Davis et al. 2010; Lihong 2016; Qun 2016; Wei et al. 2015; Xilu and Jing 2015; Yan and Qin 2015; Yixuan et al. 2019) were finally included in the systematic review and meta-analysis. A total of 1316 people were involved; six studies were published in Chinese (Lihong 2016; Qun 2016; Wei et al. 2015; Xilu and Jing 2015; Yan and Qin 2015; Yixuan et al. 2019), and one study was published in English (Davis et al. 2010); these were all case–control studies. Of these, six papers (Davis et al. 2010; Lihong 2016; Wei et al. 2015; Xilu and Jing 2015; Yan and Qin 2015; Yixuan et al. 2019) provided data related to anxiety in patients with POI, and five papers (Davis et al. 2010; Qun 2016; Wei et al. 2015; Yan and Qin 2015; Yixuan et al. 2019) provided data relating to depression in patients with POI. Four articles (Davis et al. 2010; Lihong 2016; Xilu and Jing 2015; Yixuan et al. 2019) focused on anxiety and depression diagnosis using professional clinical scales, such as the State-Trait Anxiety Inventory (STAI), Center of Epidemiologic Studies Depression Scale (CES-D), Self-Rating Anxiety Scale (SAS), and the Self-Rating Depression Scale (SDS); and the other three articles (Qun 2016; Wei et al. 2015; Yan and Qin 2015) involved self-reporting. In addition, four articles (Davis et al. 2010; Lihong 2016; Qun 2016; Yan and Qin 2015) adjusted for confounding factors while the other three did not (Wei et al. 2015; Xilu and Jing 2015; Yixuan et al. 2019). Six studies (Davis et al. 2010; Lihong 2016; Qun 2016; Xilu and Jing 2015; Yan and Qin 2015; Yixuan et al. 2019) provided detailed odd ratios (ORs) and 95% confidence intervals (CIs). For one article (Wei et al. 2015), we calculated ORs and 95% CIs based on the total number of cases and controls. All of the included literature had NOS scores above seven and high evidence of literature quality. Table 1 shows the basic information relating to the contents.

### Overall estimates

**Fig. 2:**
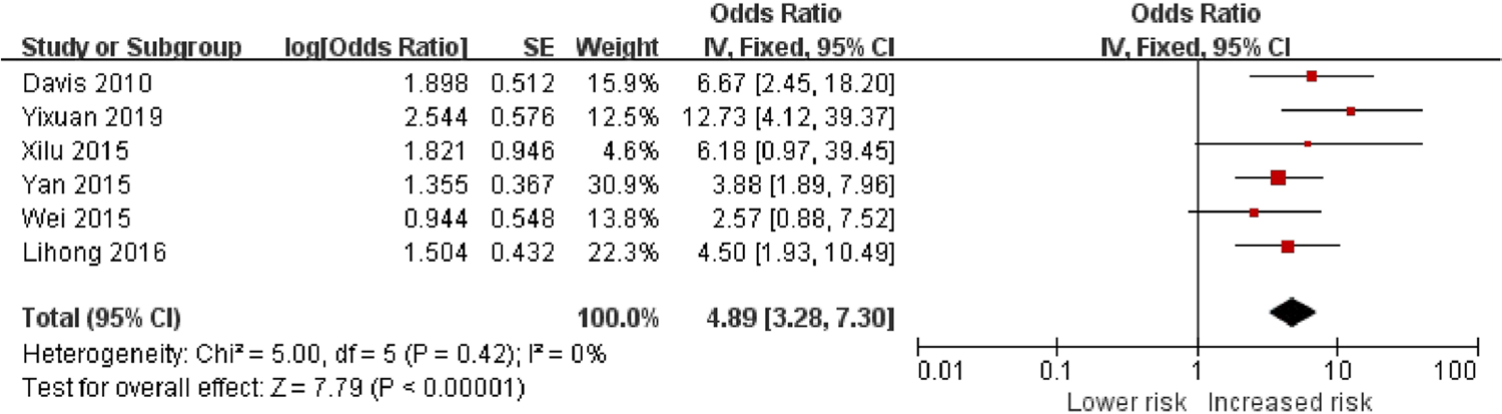
Forest plot of the risk of anxiety in POI

**Fig. 3:**
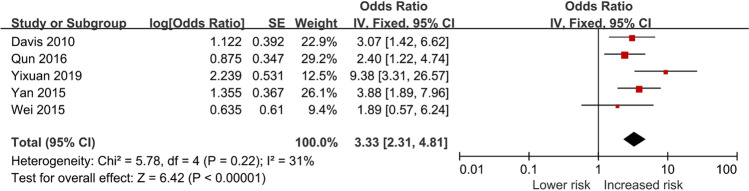
Forest plot of the risk of depression in POI

Figure 2 shows that exposure to POI increases the risk of anxiety by 4.89-fold compared with the controls (OR = 4.89, 95% CI = 3.28–7.30, *P* < 0.01). We finally included a summary of six papers and tested the heterogeneity of the integrated data using a fixed-effects model (*P* = 0.42, *I*^2^ = 0% < 50%); the data were homogenous. Figure 3 indicates that the risk of depression was elevated by 3.33-fold when associated with POI when compared to controls (OR = 3.33, 95% CI = 2.31–4.81, *P* < 0.01); this was based on the results from a fixed-effects model performed on the final inclusion of five papers. Heterogeneity tests revealed homogeneity (*P* = 0.22 and *I*^2^ = 31% < 50%).

### Subgroup analysis

We performed a subgroup analysis for the mode of diagnosis, region, and whether to adjust for confounding factors. Tables 2 and 3 show the results of the subgroup analysis. These findings suggested that POI was a potential risk factor for anxiety and depression in women.

**Table 3:** Results of subgroups analyses of anxiety-related ORs and 95% CIs

Total and subgroups	Studies	Heterogeneity (I^2^, P)	Model	OR (95% CIs)	*P*
Total	6	0, 0.42	Fixed	4.89(3.28−7.30)	< 0.001
Assessment of anxiety					
STAI	3	0, 0.83	Fixed	5.39(2.93−9.93)	< 0.001
SAS	1	/	Fixed	12.73(4.12−39.37)	< 0.001
Self-report	2	0, 0.53	Fixed	3.41(1.88−6.21)	< 0.001
Ethnicity					
America	1	/	Fixed	6.67(2.45−18.20)	< 0.001
China	5	12, 0.33	Fixed	4.62(2.99−7.14)	< 0.001
Adjustment of confounding factors					
Yes	3	0, 0.69	Fixed	4.61(2.85−7.46)	< 0.001
No	3	51, 0.13	Random	5.74(1.97−16.74)	0.001

*STAI* the state anxiety subscale of the State-Trait Anxiety Inventory; *SAS* Self-rating Anxiety Scale; *OR* odds ratio; *CI* confidence interval

There were some differences in the risk of anxiety and depression among patients with POI in different regions. The risk of anxiety onset in women with POI in Chinese regions was 4.62-fold higher (OR = 4.62, 95% CI = 2.99–7.14, *P* < 0.01) with low heterogeneity (*I*^2^ = 12%,* P* = 0.33). Comparatively, US patients with POI had a 2.05-fold increased risk of anxiety. The risk of developing anxiety in patients with POI was 6.67-fold higher in the US (OR = 6.67, 95% CI = 2.45–18.20, *P* < 0.01). However, the risk of depression among patients with POI was similar when compared between China and the USA (China: OR = 3.41, 95% CI = 2.25–5.19, *P* < 0.01; USA: OR = 3.07, 95% CI = 1.42–6.62, *P* = 0.004). The combined fixed-effects model showed low levels of heterogeneity.

Different diagnostic approaches to anxiety and depression lead to different results. In the original article, three methods were described for diagnosing depression, for example, the CES-D scale (OR = 3.07, 95% CI = 1.42–6.62, *P* = 0.004), the SDS scale (OR = 9.38, 95% CI = 3.31–26.57, *P* < 0.01), and self-reported anxiety (OR = 2.81, 95% CI = 1.78–4.44, *P* < 0.01). Similarly, the results also differed by anxiety diagnosis when pooled: SAS (OR = 12.73, 95% CI = 4.12–39.37, *P* < 0.01), STAI (OR = 5.39, 95% CI = 2.93–9.93, *P* < 0.01), and self-reporting (OR = 3.41, 95% CI = 1.88–6.21, *P* < 0.01). Professional scales measured higher ORs than self-reports, regardless of whether the risk was for anxiety or depression.

In addition, the original article was adjusted for covariates; this affected the pooled odds ratios. After adjusting for confounders, the OR for anxiety was 4.61 (OR = 4.61, 95% CI = 2.58–7.46, *P* < 0.01), and the risk of developing depression was 3.03-fold higher (OR = 3.03, 95% CI = 2.00–4.59, *P* < 0.01). However, the ORs of non-adjusted confounders were higher than those of adjusted confounders (anxiety: OR = 5.74, 95% CI = 1.97–16.74, *P* = 0.001; depression: OR = 4.33, 95% CI = 0.90–20.82, *P* = 0.07).

### Bias assessment

**Fig. 4:**
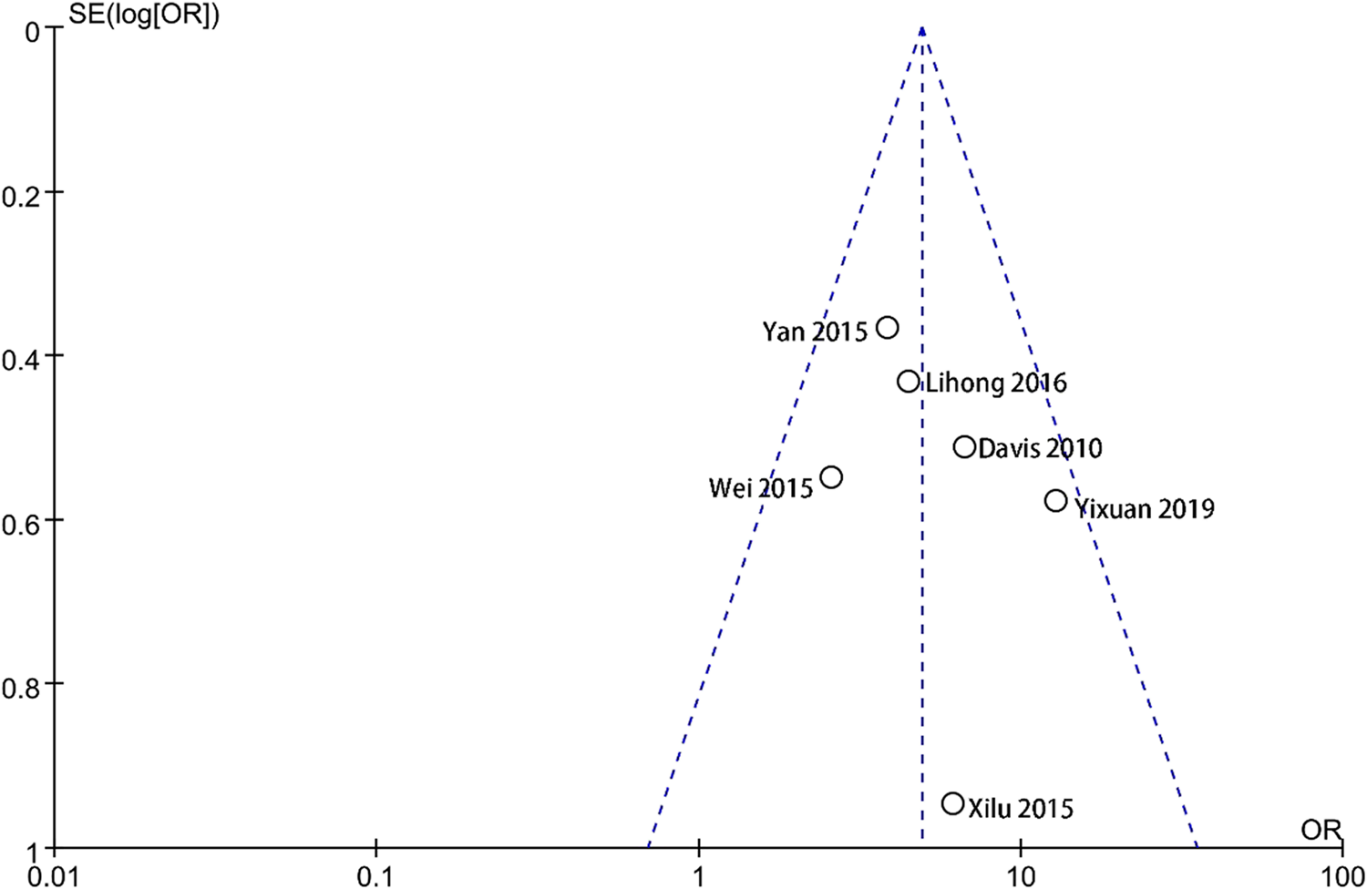
Funnel plots of the risk of anxiety in POI

**Fig. 5:**
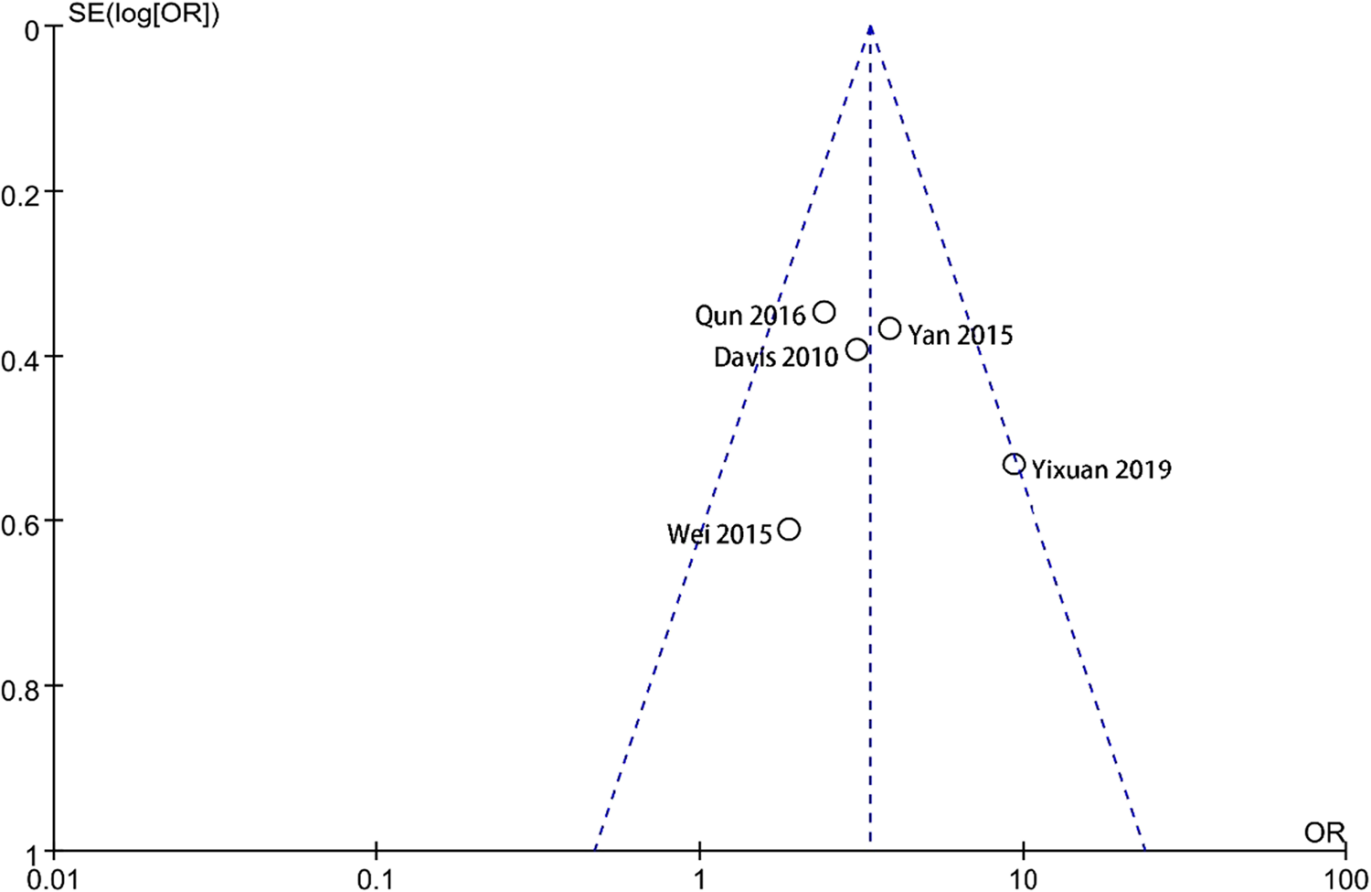
Funnel plots of the risk of depression in POI

The funnel plot of the literature on depressive symptoms with POI shows bilateral symmetry and no publication bias (Fig. 4). However, the funnel plot analysis of the six papers reporting anxiety with POI is found to be asymmetrical (Fig. 5). The main reason is the limited number of studies, resulting in an asymmetric funnel plot.

## Discussion

### Analysis of results

The purpose of this study was to investigate the risk of anxiety and depression prevalence in patients with POI. Our findings showed that the prevalence of anxiety and depression in patients with POI was 4.89-fold and 3.33-fold higher than those in healthy controls, respectively. According to one study, women with polycystic ovary syndrome (PCOS) had a 6.3-fold and 3.78-fold increased odds of anxiety and depressive symptoms, respectively, when compared to women without PCOS (Cooney et al. 2017). Perimenopausal women also had a two-fold increased risk of depression compared to menopause (De Kruif et al. 2016). Studies have different baseline information and cannot directly compare incidence rates. However, these data indicated that physical illness in women could increase the risk of developing mental illness. This observation needs to be taken seriously.

The specific etiology of anxiety or depression in patients with POI remains unclear. However, potential factors may be related to neuroendocrine regulation, cytokines, and psychosocial factors. Research has shown that patients with POI are characterized by estrogen deficiency and high gonadotropin levels. However, high levels of follicle-stimulating hormone (FSH), along with the vasodilation and somatic symptoms of perimenopausal women, are known to increase the risk of anxiety and depression (Yanyan 2012). On the other hand, estrogen deficiency and the development of mood disorders in women are significantly related (Payne 2003). A reduction in the levels of estrogen can lead to a relative increase in the levels of FSH and LH, thus contributing to an imbalance in the hypothalamic-pituitary–gonadal (HPG) axis and HPA axis dysregulation (Gordon et al. 2016). Reduced levels of estrogen are associated with neuroendocrine disorders and could be implicated in the impaired synthesis, decreased metabolism, and reduced receptor activity of classical neurotransmitters (e.g., 5-hydroxytryptamine, dopamine, and norepinephrine) (Gava et al. 2019), which may result in patients with POI being more susceptible to mood disorders. Second, the pathogenesis of POI with anxiety or depression may be relevant to the levels of transforming growth factor-β (TGF-β) and interferon gamma (IFN-γ). TGF-β and IFN-γ are associated with patients who suffer from premature ovarian failure (Jian et al. 2020; Mantawy et al. 2019). Moreover, levels of TGF-β and IFN-γ are closely related to depression (Miller 2010; Myint et al. 2005). Patients with psychological abnormalities such as anxiety and depression are accompanied by immune abnormalities and activation of the inflammatory response system. Third, POI with anxiety and depression may be associated with psychosocial factors. A previous study showed that (Yusuf 2016) the extent of anxiety and depression in infertile patients is significantly higher than the prevalence in fertile females. POI not only raises a woman’s risk of bone loss and cardiovascular disease but also increases the burden of fertility for women who want to have children. As a result, infertile women who suffer from POI are under greater levels of stress. Moreover, the public stigma of this disease is aggravated by the subfertility caused by POI (Colhoun et al. 2018; Golezar et al. 2020). These factors may be critical to understanding the greater vulnerability of patients with POI to depression and anxiety disorders.

Comorbid depression or anxiety with POI often occurs in the clinical setting. Therefore, these diseases may have a common etiology. It is well known that emotional disorders are closely associated with the pathogenesis of POI. The function of the HPG axis is linked to POI (Da Costa et al. 2021). Anxiety and depression are also closely related to the HPG axis (Dwyer et al. 2020). POI with metabolic and reproductive problems, as well as chronic and incurable conditions, may cause problems with depression and anxiety. Meanwhile, mood disorders could induce amenorrhea and ovarian dysfunction. Chronic negative emotions activate the HPA axis, thus leading to the secretion of large amounts of cortisol, which inhibits the pulsatile release rhythm of the hypothalamic gonadotropin-releasing hormone (GnRH) (Breen et al. 2005; Zefferino et al. 2021). Furthermore, mental stress induces the secretin of beta-endorphin secretion in the hypothalamus and pituitary gland, thus affecting the secretion of gonadotropins (O’Connor et al. 2011). As a result, mental stress exerts an influence on ovarian function, which may lead to premature ovarian failure.

### Subgroup analyses and bias

We conducted a subgroup analysis to investigate the effects of different subgroups on our findings. Differences in the development of POI with anxiety and depression between the USA and China. In China, the risk of anxiety and depression was 4.62- and 3.14-fold higher in patients with POI when compared with the healthy population. The risk of anxiety and depression for patients with POI in the USA was previously reported to be 6.67 times and 3.07 times, respectively. The risk of developing anxiety in patients with POI was reported to be higher in the USA than in China. There are several reasons for this. Firstly, the mental health care resources and cultural differences between China and the USA may exert an influence on the diagnostic rate of mental illness. Secondly, self-reported results are not accurately diagnosed due to the stigmatization associated with the disease; furthermore, these self-reported studies all originated from China. Thirdly, there was only one study data from the USA in the subgroup analysis. Therefore, the results arising from the subgroup analysis may be biased. Further studies are now needed to verify the source of the difference in the prevalence of anxiety in the USA and China. All these factors may contribute to the differences in the risk of anxiety and depression occurrence in patients with POI when compared between the USA and China.

Professional scales indicated higher ORs than self-reporting. The difficulty in differentiating between anxiety and depression will result in the underdiagnosis and misdiagnosis of anxiety and depression. Therefore, more specialized scales are required rather than self-reports to diagnose anxiety and depression. In addition, confounding factors are also important sources of bias in clinical research. Three of the original studies did not adjust for covariates; this may have led to notable bias in the findings. Covariates should be adjusted to ensure the authenticity and reliability of original studies.

### Clinical and research applications

There is a clear need to take appropriate measures based on the high prevalence of depression and anxiety in POI. Firstly, physicians need to assess the mental health and follow up patients with POI as necessary. This is because patients with POI not only have abnormal menstruation and infertility, but they may also have mental disorders. Hormone replacement therapy may alleviate anxiety and depressive symptoms in patients with POI, but it remains limited. Secondly, patients with mild mood disorders can also consider both non-pharmacologic (i.e., exercise, and dietary supplements) and hormonal strategies. Patients with POI with moderate to severe psychological disorders should be recommended multidisciplinary approaches, such as combined hormone replacement therapy and psychotherapies (i.e., cognitive therapy, transcranial magnetic stimulation, and selective serotonin reuptake inhibitor). Patients with POI unsuitable for hormone therapy can receive complementary and alternative therapies (e.g., herbal medicine and acupuncture) and psychotherapy in combination. Thirdly, public health authorities may pay more attention to publicizing knowledge related to POI and mental health. On one hand, it is important to raise public awareness with regard to the prevention of POI and relevant mental illnesses. Furthermore, this strategy could eliminate the stigmatization suffered by patients with POI. In addition, these methods will improve patient compliance and alleviate adverse emotions, as well as improve quality of life.

Importantly, future studies should focus on the longitudinal follow-up of patients with POI. The long-term effects of POI treatment on the symptoms of depression and anxiety in patients with POI need to be investigated further. Therefore, prospective cohort studies should design in a standardized manner. In addition, we need to further investigate the etiology and pathogenesis of POI with mood disorders. Furthermore, we should conduct randomized controlled trials or real-world studies of integrative multidisciplinary interventions for this population so as to provide patients with more standardized and comprehensive individualized treatments.

### Advantages and disadvantages

To the best of our knowledge, this is the first meta-analysis of the relationship between POI and depression or anxiety. This research represents a stepping stone for future research with regard to investigating POI and supportive interventions for this specific group of patients. The study also highlights the importance of supporting POI during this stressful transition period. In particular, clinical treatment should be individualized and changed from a single-disciplinary model to a multi-disciplinary team (MDT) for patients with POI. In addition, this study controlled potential sources of clinical heterogeneity by establishing strict inclusion and exclusion criteria. Furthermore, sources of methodological heterogeneity were minimized. We scored all of the included articles with a mean NOS score greater than 7 and a high quality. Moreover, we tested the meta-analysis for statistical heterogeneity and selected a suitable effects model to integrate the data. Publication bias was checked by generating a funnel plot. These methods were used to minimize heterogeneity and increase the reliability and validity of the outcomes. The findings of this study provide new evidence for the clinical management of patients with POI combined with anxiety or depression.

Our study has certain limitations that need to be considered. Firstly, the pooled literature originated mainly from China; thus, the results of subgroup analysis from different countries and regions may be biased. Further investigations of anxiety and depression in patients with POI in non-Chinese regions are now required to gain a better and more comprehensive understanding of the impact of POI on patients’ mental health. Secondly, we were not able to assess other potential factors contributing to depressive and anxiety symptoms in patients with POI, such as infertility factors, hormone levels, disease duration, and treatment regimen. We will continue to add these data as the study progresses in the future.

## Conclusion

This meta-analysis revealed that POI significantly increased the risk of anxiety and depression. Understanding the epidemiology of psychological disorders among patients with POI is critical. Therefore, we suggest that treating mental illnesses should be an important part of prevention programs for POI. In the future, the mental health of patients with POI should be considered in more detail. To provide more targeted interventions and treatments, it is important that we focus on the underpinning mechanisms contributing to the enhanced risk of depression and anxiety in patients with POI.

## Supplementary Information

Below is the link to the electronic supplementary material.

Supplementary file1 (PDF 186 KB)
