# Supplementary material for: The risk of depressive and anxiety symptoms in women with premature ovarian insufficiency: a systematic review and meta-analysis
Source: Arch Womens Ment Health. 2023 Jan 27;26(1):1–10. doi: 10.1007/s00737-022-01289-7 (PMC9908676; doi:10.1007/s00737-022-01289-7)
Supplement: Supplementary file 1 — Supplementary file1 (PDF 186 KB) [file 737_2022_1289_MOESM1_ESM.pdf]

**Appendix 1**　Strategies and evidence of retrieval

1、China National Knowledge Infrastructure (CNKI)：(SU=’原发性卵巢功能不全’+’早发性卵巢功能不全’ OR TKA='POI'+'POF'+’抗促性腺素卵巢综合征’+’卵巢功能早衰’+’抗拒性卵巢综合征’+’卵巢早衰’+’卵巢功能低下’+’卵巢功能受损’+’卵巢功能衰退’+’绝经，提早’) AND( SU=’抑郁’+’抑郁症’+’焦虑’+’焦虑症’ OR TKA=’抑郁症状’+’抑郁症，内因性’+’抑郁症，神经官能性’+’抑郁症，单相’+’抑郁综合征’+’忧郁症’+’神经症，抑郁性’+’单相抑郁症’+’抑郁障碍’+’焦虑症’+’焦虑性神经症’+’焦虑状态，神经官能症性’+’神经症，焦虑性’+’焦虑症状’+’焦虑障碍’)

2、Wan Fang Data：(主题:("原发性卵巢功能不全" or "早发性卵巢功能不全") or 题名或关键词:("POI" or "POF" or "抗促性腺素卵巢综合征" or "卵巢功能早衰" or "抗拒性卵巢综合征" or "卵巢早衰" or "卵巢功能低下" or "卵巢功能受损" or "卵巢功能衰退" or "绝经，提早")) and (主题:("抑郁" or "抑郁症" or "焦虑" or "焦虑症") or 题名或关键词:("抑郁症状" or "抑郁症，内因性" or "抑郁症，神经官能性" or "抑郁症，单相" or "抑郁综合征" or "忧郁症" or "神经症，抑郁性" or "单相抑郁症" or "抑郁障碍" or "焦虑性神经症" or "焦虑状态，神经官能症性" or "神经症，焦虑性" or "焦虑症状" or "焦虑障碍"))

3、Chongqing VIP Information (CQVIP)：(M=(原发性卵巢功能不全 + 早发性卵巢功能不全) OR R=(POI + POF + 抗促性腺素卵巢综合征 + 卵巢功能早衰 + 抗拒性卵巢综合征 + 卵巢早衰 + 卵巢功能低下+ 卵巢功能受损 + 卵巢功能衰退 + 绝经，提早)) AND (M=(抑郁 + 抑郁症 + 焦虑 + 焦虑症) OR R=(抑郁症状 + 抑郁症，内因性 + 抑郁症，神经官能性 + 抑郁症，单相 + 抑郁综合征 + 忧郁症 + 神经症，抑郁性 + 单相抑郁症 + 抑郁障碍 + 焦虑性神经症 + 焦虑状态，神经官能症性 + 神经症，焦虑性 + 焦虑症状 + 焦虑障碍))

4、SinoMed：("原发性卵巢功能不全"[常用字段:智能] OR "POI"[常用字段:智能] OR "POF"[常用字段:智能] OR "抗促性腺素卵巢综合征"[常用字段:智能] OR "卵巢功能早衰"[常用字段:智能] OR "抗拒性卵巢综合征"[常用字段:智能] OR "卵巢早衰"[常用字段:智能] OR "早发性卵巢功能不全"[常用字段:智能] OR "卵巢功能低下"[常用字段:智能] OR "卵巢功能受损"[常用字段:智能] OR "卵巢功能衰退"[常用字段:智能] OR "绝经，提早"[常用字段:智能]) AND ("抑郁"[常用字段:智能] OR "抑郁症状"[常用字段:智能] OR "抑郁症"[常用字段:智能] OR "抑郁症，内因性"[常用字段:智能] OR "抑郁症，神经官能性"[常用字段:智能] OR "抑郁症，单相"[常用字段:智能] OR "抑郁综合征"[常用字段:智能] OR "忧郁症"[常用字段:智能] OR "神经症，抑郁性"[常用字段:智能] OR "单相抑郁症"[常用字段:智能] OR "抑郁障碍"[常用字段:智能] OR "焦虑"[常用字段:智能] OR "焦虑症"[常用字段:智能] OR "焦虑性神经症"[常用字段:智能] OR "焦虑状态，神经官能症性"[常用字段:智能] OR "神经症，焦虑性"[常用字段:智能] OR "焦虑症状"[常用字段:智能] OR "焦虑障碍"[常用字段:智能])

5、Pubmed：((((((((((((((((((((((Menopause, Premature [Title/Abstract])) OR (Premature Menopause[Title/Abstract])) OR (Primary Ovarian Insufficiency[Title/Abstract])) OR (Ovarian Insufficiency, Primary[Title/Abstract])) OR (Ovarian Failure, Premature[Title/Abstract])) OR (Premature Ovarian Failure[Title/Abstract])) OR (Gonadotropin-Resistant Ovary Syndrome[Title/Abstract])) OR (Gonadotropin Resistant Ovary Syndrome[Title/Abstract])) OR (Resistant Ovary Syndrome[Title/Abstract])) OR (Hypergonadotropic Ovarian Failure, X-

Linked[Title/Abstract])) OR (Hypergonadotropic Ovarian Failure, X Linked[Title/Abstract])) OR (X-Linked Hypergonadotropic Ovarian Failure[Title/Abstract])) OR (X Linked Hypergonadotropic Ovarian Failure[Title/Abstract])) OR (Premature Ovarian Failure, X-Linked[Title/Abstract])) OR (Premature Ovarian Failure, X Linked[Title/Abstract])) OR (Fragile X-Associated Primary Ovarian Insufficiency[Title/Abstract])) OR (Fragile X Associated Primary Ovarian Insufficiency[Title/Abstract])) OR (Fragile X Premature Ovarian Failure[Title/Abstract])) OR (FMR1-Related Primary Ovarian Insufficiency[Title/Abstract])) OR (FMR1 Related Primary Ovarian Insufficiency[Title/Abstract])) OR (Primary Ovarian Insufficiency, Fragile X-Associated[Title/Abstract])) OR (Primary Ovarian Insufficiency, Fragile X Associated[Title/Abstract])) OR (Premature Ovarian Failure 1[Title/Abstract])) OR (premature ovarian failure[Title/Abstract])) AND ((((((((((((((((((((((((((((((((((((Depression[Title/Abstract])) OR (Depressions[Title/Abstract])) OR (Depressive Symptoms[Title/Abstract])) OR (Depressive Symptom[Title/Abstract])) OR (Symptom, Depressive[Title/Abstract])) OR (Symptoms, Depressive[Title/Abstract])) OR (Emotional Depression[Title/Abstract])) OR (Depression, Emotional[Title/Abstract])) OR (Depressions, Emotional[Title/Abstract])) OR (Emotional Depressions[Title/Abstract])) OR (Depressive Disorder[Title/Abstract])) OR (Depressive Disorders[Title/Abstract])) OR (Disorder, Depressive[Title/Abstract])) OR (Disorders, Depressive[Title/Abstract])) OR (Neurosis, Depressive[Title/Abstract])) OR (Depressive Neuroses[Title/Abstract])) OR (Depressive Neurosis[Title/Abstract])) OR (Neuroses, Depressive[Title/Abstract])) OR (Depression, Endogenous[Title/Abstract])) OR (Depressions, Endogenous[Title/Abstract])) OR (Endogenous Depression[Title/Abstract])) OR (Endogenous Depressions[Title/Abstract])) OR (Depressive Syndrome[Title/Abstract])) OR (Depressive Syndromes[Title/Abstract])) OR (Syndrome, Depressive[Title/Abstract])) OR (Syndromes, Depressive[Title/Abstract])) OR (Depression, Neurotic[Title/Abstract])) OR (Depressions, Neurotic[Title/Abstract])) OR (Neurotic Depression[Title/Abstract])) OR (Neurotic Depressions[Title/Abstract])) OR (Melancholia[Title/Abstract])) OR (Melancholias[Title/Abstract])) OR (Unipolar Depression[Title/Abstract])) OR (Depression, Unipolar[Title/Abstract])) OR (Depressions, Unipolar[Title/Abstract])) OR (Unipolar Depressions[Title/Abstract])) OR ((((((((((((((((((((anxiety[Title/Abstract])) OR (Angst[Title/Abstract])) OR (Nervousness[Title/Abstract])) OR (Hypervigilance[Title/Abstract])) OR (Anxiousness[Title/Abstract])) OR (Social Anxiety[Title/Abstract])) OR (Anxieties, Social[Title/Abstract])) OR (Anxiety, Social[Title/Abstract])) OR (Social Anxieties[Title/Abstract])) OR (Anxiety Disorders[Title/Abstract])) OR (Anxiety Disorder[Title/Abstract])) OR (Disorder, Anxiety[Title/Abstract])) OR (Disorders, Anxiety[Title/Abstract])) OR (Neuroses, Anxiety[Title/Abstract])) OR (Anxiety Neuroses[Title/Abstract])) OR (Anxiety States, Neurotic[Title/Abstract])) OR (Anxiety State, Neurotic[Title/Abstract])) OR (anxiety syptom[Title/Abstract])) OR (anxiety disease[Title/Abstract])) OR (Angst disease[Title/Abstract])))

6、Embase:('early menopause':ab,ti OR 'premature menopause':ab,ti OR 'ovarian insufficiency, primary':ab,ti OR 'ovarian failure, premature':ab,ti OR 'premature ovarian failure':ab,ti OR 'gonadotropin-resistant ovary syndrome':ab,ti OR 'gonadotropin resistant ovary syndrome':ab,ti OR 'resistant ovary syndrome':ab,ti OR 'hypergonadotropic ovarian failure, x-linked':ab,ti OR 'hypergonadotropic ovarian failure, x linked':ab,ti OR 'x-linked hypergonadotropic ovarian failure':ab,ti OR 'x linked hypergonadotropic ovarian failure':ab,ti OR 'premature ovarian failure, x-

linked':ab,ti OR 'premature ovarian failure, x linked':ab,ti OR 'fragile x-associated primary ovarian insufficiency':ab,ti OR 'fragile x associated primary ovarian insufficiency':ab,ti OR 'fragile x premature ovarian failure':ab,ti OR 'fmr1-related primary ovarian insufficiency':ab,ti OR 'fmr1 related primary ovarian insufficiency':ab,ti OR 'primary ovarian insufficiency, fragile x-associated':ab,ti OR 'primary ovarian insufficiency, fragile x associated':ab,ti OR 'premature ovarian failure 1':ab,ti) AND ('depression':ab,ti OR depressions:ab,ti OR 'depressive symptoms':ab,ti OR 'depressive symptom':ab,ti OR 'symptom, depressive':ab,ti OR 'symptoms, depressive':ab,ti OR 'emotional depression':ab,ti OR 'depression, emotional':ab,ti OR 'depressions, emotional':ab,ti OR 'emotional depressions':ab,ti OR 'depressive disorder':ab,ti OR 'depressive disorders':ab,ti OR 'disorder, depressive':ab,ti OR 'disorders, depressive':ab,ti OR 'neurosis, depressive':ab,ti OR 'depressive neuroses':ab,ti OR 'depressive neurosis':ab,ti OR 'neuroses, depressive':ab,ti OR 'depression, endogenous':ab,ti OR 'depressions, endogenous':ab,ti OR 'endogenous depression':ab,ti OR 'endogenous depressions':ab,ti OR 'depressive syndrome':ab,ti OR 'depressive syndromes':ab,ti OR 'syndrome, depressive':ab,ti OR 'syndromes, depressive':ab,ti OR 'depression, neurotic':ab,ti OR 'depressions, neurotic':ab,ti OR 'neurotic depression':ab,ti OR 'neurotic depressions':ab,ti OR melancholia:ab,ti OR melancholias:ab,ti OR 'unipolar depression':ab,ti OR 'depression, unipolar':ab,ti OR 'depressions, unipolar':ab,ti OR 'unipolar depressions':ab,ti OR 'anxiety':ab,ti OR angst:ab,ti OR nervousness:ab,ti OR hypervigilance:ab,ti OR anxiousness:ab,ti OR 'social anxiety':ab,ti OR 'anxieties, social':ab,ti OR 'anxiety, social':ab,ti OR 'social anxieties':ab,ti OR 'anxiety disorder':ab,ti OR 'anxiety disorders':ab,ti OR 'disorder, anxiety':ab,ti OR 'disorders, anxiety':ab,ti OR 'neuroses, anxiety':ab,ti OR 'anxiety neuroses':ab,ti OR 'anxiety states, neurotic':ab,ti OR 'anxiety state, neurotic':ab,ti OR 'neurotic anxiety state':ab,ti OR 'neurotic anxiety states':ab,ti OR 'state, neurotic anxiety':ab,ti OR 'states, neurotic anxiety':ab,ti OR 'anxiety syptom':ab,ti OR 'anxiety disease':ab,ti OR 'angst disease':ab,ti)

7、Web of science：(TS=(Menopause, Premature OR Primary Ovarian Insufficiency OR premature ovarian failure) OR AB=(Premature Menopause OR Ovarian Insufficiency, Primary OR Ovarian Failure, Premature OR Premature Ovarian Failure OR Gonadotropin-Resistant Ovary Syndrome OR Gonadotropin Resistant Ovary Syndrome OR Resistant Ovary Syndrome OR Hypergonadotropic Ovarian Failure, X-Linked Hypergonadotropic Ovarian Failure, X Linked OR X-Linked Hypergonadotropic Ovarian Failure OR X Linked Hypergonadotropic Ovarian Failure OR Premature Ovarian Failure, X-Linked OR Premature Ovarian Failure, X Linked OR Fragile X-Associated Primary Ovarian Insufficiency OR Fragile X Associated Primary Ovarian Insufficiency OR Fragile X Premature Ovarian Failure OR FMR1-Related Primary Ovarian Insufficiency OR FMR1 Related Primary Ovarian Insufficiency OR Primary Ovarian Insufficiency, Fragile X-Associated OR Primary Ovarian Insufficiency, Fragile X Associated OR Premature Ovarian Failure 1)) AND (TS=(Depression OR Depressive Disorder OR anxiety OR Anxiety Disorders) OR AB=(Depressions OR Depressive Symptoms OR Depressive Symptom OR Symptom, Depressive OR Symptoms, Depressive OR Emotional Depression OR Depression, Emotional OR Depressions, Emotional OR Emotional Depressions OR Depressive Disorders OR Disorder, Depressive OR Disorders, Depressive OR Neurosis, Depressive OR Depressive Neuroses OR Depressive Neurosis OR Neuroses, Depressive OR Depression, Endogenous OR Depressions, Endogenous OR Endogenous Depression OR Endogenous Depressions OR Depressive Syndrome OR Depressive Syndromes OR Syndrome, Depressive OR Syndromes, Depressive OR Depression, Neurotic OR Depressions, Neurotic OR Neurotic Depression

OR Neurotic Depressions OR Melancholia OR Melancholias OR Unipolar Depression OR Depression, Unipolar OR Depressions, Unipolar OR Unipolar Depressions OR Angst OR Nervousness OR Hypervigilance OR Anxiousness OR Social Anxiety OR Anxieties, Social OR Anxiety, Social OR Social Anxieties OR Anxiety Disorder OR Disorder, Anxiety OR Disorders, Anxiety OR Neuroses, Anxiety OR Anxiety Neuroses OR Anxiety States, Neurotic OR Anxiety State, Neurotic OR Neurotic Anxiety State OR Neurotic Anxiety States OR State, Neurotic Anxiety OR States, Neurotic Anxiety OR anxiety syptom OR anxiety disease OR Angst disease))

8、Cochrane Library：

ID Search

#1 MeSH descriptor: [Menopause, Premature] 3 tree(s) exploded

#2 MeSH descriptor: [Primary Ovarian Insufficiency] 2 tree(s) exploded

#3 (Premature Menopause):ab,ti,kw OR (Ovarian Insufficiency, Primary):ab,ti,kw OR (Ovarian Failure, Premature):ab,ti,kw OR (Premature Ovarian Failure):ab,ti,kw OR (Gonadotropin-Resistant Ovary Syndrome):ab,ti,kw OR (Gonadotropin Resistant Ovary Syndrome):ab,ti,kw OR (Resistant Ovary Syndrome):ab,ti,kw OR (Hypergonadotropic Ovarian Failure, X-Linked):ab,ti,kw OR (Hypergonadotropic Ovarian Failure, X Linked):ab,ti,kw OR (X-Linked Hypergonadotropic Ovarian Failure):ab,ti,kw OR (X Linked Hypergonadotropic Ovarian Failure):ab,ti,kw OR (Premature Ovarian Failure, X-Linked):ab,ti,kw OR (Premature Ovarian Failure, X Linked):ab,ti,kw OR (Fragile X-Associated Primary Ovarian Insufficiency):ab,ti,kw OR (Fragile X Associated Primary Ovarian Insufficiency):ab,ti,kw OR (Fragile X Premature Ovarian Failure):ab,ti,kw OR (Primary Ovarian Insufficiency, Fragile X-Associated):ab,ti,kw OR (Primary Ovarian Insufficiency, Fragile X Associated):ab,ti,kw OR (Premature Ovarian Failure 1):ab,ti,kw OR (premature ovarian failure):ab,ti,kw

#4 #1 OR #2 OR #3

#5 MeSH descriptor: [Depression] 1 tree(s) exploded

#6 MeSH descriptor: [Depressive Disorder] explode all trees

#7 (Depressions):ab,ti,kw OR (Depressive Symptoms):ab,ti,kw OR (Depressive Symptom):ab,ti,kw OR (Symptom, Depressive):ab,ti,kw OR (Symptoms, Depressive):ab,ti,kw OR (Emotional Depression):ab,ti,kw OR (Depression, Emotional):ab,ti,kw OR (Depressions, Emotional):ab,ti,kw OR (Emotional Depressions):ab,ti,kw OR (Depressive Disorders):ab,ti,kw OR (Disorder, Depressive):ab,ti,kw OR (Disorders, Depressive):ab,ti,kw OR (Neurosis, Depressive):ab,ti,kw OR (Depressive Neuroses):ab,ti,kw OR (Depressive Neurosis):ab,ti,kw OR (Neuroses, Depressive):ab,ti,kw OR (Depression, Endogenous):ab,ti,kw OR (Depressions, Endogenous):ab,ti,kw OR (Endogenous Depression):ab,ti,kw OR (Endogenous Depressions):ab,ti,kw OR (Depressive Syndrome):ab,ti,kw OR (Depressive Syndromes):ab,ti,kw OR (Syndrome, Depressive):ab,ti,kw OR (Syndromes, Depressive):ab,ti,kw OR (Depression, Neurotic):ab,ti,kw OR (Depressions, Neurotic):ab,ti,kw OR (Neurotic Depression):ab,ti,kw OR (Neurotic Depressions):ab,ti,kw OR (Melancholia):ab,ti,kw OR (Melancholias):ab,ti,kw OR (Unipolar Depression):ab,ti,kw OR (Depression, Unipolar):ab,ti,kw OR (Depressions, Unipolar):ab,ti,kw OR (Unipolar Depressions):ab,ti,kw

#8 #5 OR #6 OR #7

#9 MeSH descriptor: [Anxiety] explode all trees

#10 MeSH descriptor: [Anxiety Disorders] explode all trees

#11 (Angst):ab,ti,kw OR (Nervousness):ab,ti,kw OR (Hypervigilance):ab,ti,kw OR (Anxiousness):ab,ti,kw OR (Social Anxiety):ab,ti,kw OR (Anxieties, Social):ab,ti,kw OR (Anxiety, Social):ab,ti,kw OR (Social Anxieties):ab,ti,kw OR (Anxiety Disorder):ab,ti,kw OR (Disorder, Anxiety):ab,ti,kw OR (Disorders, Anxiety):ab,ti,kw OR (Neuroses, Anxiety):ab,ti,kw OR (Anxiety Neuroses):ab,ti,kw OR (Anxiety States, Neurotic):ab,ti,kw OR (Anxiety State, Neurotic):ab,ti,kw OR (Neurotic Anxiety State):ab,ti,kw OR (Neurotic Anxiety States):ab,ti,kw OR (State, Neurotic Anxiety):ab,ti,kw OR (States, Neurotic Anxiety):ab,ti,kw OR (anxiety syptom):ab,ti,kw OR (anxiety disease):ab,ti,kw OR (Angst disease):ab,ti,kw

#12 #9 OR #10 OR #11

#13 #8 OR #12

#14 #4 AND #13
